# Association of product of platelet and neutrophil count with monoclonal gammopathy of undetermined significance: a cross-sectional analysis of the NHANES

**DOI:** 10.1007/s44313-025-00094-2

**Published:** 2025-08-18

**Authors:** Lijie Wang, Peiyao Yang, Huijie Nan, Wenqian Li, Yuanyuan Liu, Fangfang Xu, Mingyue Shi, Yanliang Bai

**Affiliations:** 1 Department of Hematology, Henan University People’s Hospital and Henan Provincial People’s Hospital, Henan, People’s Republic of China; 2 Department of Hematology, Zhengzhou University People’s Hospital and Henan Provincial People’s Hospital, No.7 Weiwu Road, Jinshui District, Zhengzhou City, Henan Province 450003 People’s Republic of China; 3 Key Laboratory of Geriatrics, Institute of Geriatrics, Department of Geriatric Medicine, Henan Provincial People’s Hospital, People’s Hospital of Zhengzhou University, Zhengzhou, People’s Republic of China

**Keywords:** PPN inflammation index, MGUS, NHANES, Relationship, Cross-sectional study

## Abstract

**Background:**

Inflammation indices are emerging predictors of diseases. Monoclonal gammopathy of undetermined significance (MGUS) is a precancerous state and chronic inflammation may drive MGUS progression. This study aimed to evaluate the association between inflammatory markers and MGUS.

**Methods:**

Data from the National Health and Nutrition Examination Survey (NHANES) III and 1999–2004 were collected from 6,383 participants. MGUS subtypes were identified using immunofixation electrophoresis. Seven inflammatory indices [lymphocyte count (LC), neutrophil count (NC), platelet-neutrophil product (PPN), systemic immune inflammation index (SII), platelet-lymphocyte ratio (PLR), and C-reactive protein (CRP)] were calculated. Weighted multivariate regression and subgroup analyses assessed the relationships, reported as odds ratios (ORs) and 95% confidence intervals (CIs).

**Results:**

Of the 6383 patients included in the study, 157 (2.45%) underwent MGUS. There was a significant correlation trend between ln PPN level and the development of MGUS, especially at low levels (OR: 2.62, 95% CI: 1.54–4.75, *p-trend* = 0.001), while the correlation between PLR level and MGUS was not obvious. In the subgroup analysis, a significant association between PPN level and MGUS was mainly found in the overall population, female sex, non-Hispanic black, non-hypercholesterolemia, non-type 2 diabetes (T2D), high school education or above, and divorced or widowed; however, there was no significant interaction between PPN level and MGUS in each subgroup.

**Conclusion:**

PPN levels were significantly associated with MGUS development. Our study identified PPN as a novel and convenient inflammatory marker with potential clinical relevance. Although preliminary, the observed associations highlight the need for validation through longitudinal studies before considering their clinical applications.

## Introduction

Monoclonal gammopathy of undetermined significance (MGUS) is a premalignant condition, characterized by the presence of monoclonal immunoglobulins in the serum without evidence of end-organ damage such as hypercalcemia, renal insufficiency, anemia, or bone lesions [1, 2]. As an asymptomatic precursor to multiple myeloma (MM) and other plasma cell disorders, MGUS affects approximately 3% of the general population over the age of 50 years, and its prevalence increases with age [3]. Although the annual progression rate from MGUS to MM is low (approximately 1%), the identification of modifiable risk factors associated with MGUS remains a critical area of research as it may provide insights into early interventions to reduce the burden of plasma cell dyscrasias [4].

Inflammation has long been implicated in the pathogenesis of hematological malignancies, including MM [5]. However, the relationship between systemic inflammation and prevalence of MGUS, distinct from its progression to malignancy, has not been thoroughly investigated. Chronic inflammation may influence clonal expansion of plasma cells through immune dysregulation, cytokine signaling, or alterations in the bone marrow microenvironment (BMME) [6]. For instance, pro-inflammatory cytokines such as interleukin-6 (IL-6) and tumor necrosis factor-alpha (TNF-α) promote plasma cell survival and proliferation, potentially contributing to the establishment of monoclonal gammopathy [7]. In addition, changes in BMME may play a key role in MM evolution through a progressive shift towards a pro-inflammatory and immunosuppressive shape, which may drive cancer progression as well as clonal plasma cell migration, proliferation, survival, and drug resistance [8, 9]. However, population-based studies evaluating the association between systemic inflammatory markers and MGUS are limited. It may only reflect some inflammatory pathways or may be susceptible to potential associations, such as age and comorbidities.

Peripheral blood components, including leukocytes, neutrophils, lymphocytes, and platelets, play pivotal roles in inflammatory responses. These parameters are readily obtained from routine blood tests. Despite their limited diagnostic specificity, these biomarkers offer advantages, such as low cost, high reproducibility, minimal invasiveness, and broad clinical acceptance. Emerging systemic inflammatory markers derived from peripheral blood cell counts, such as platelet-neutrophil product (PPN), platelet-to-lymphocyte ratio (PLR), systemic immune-inflammation index (SII), and C-reactive Protein (CRP), are increasingly used to assess disease susceptibility and are closely associated with clinical prognosis. PPN represents a novel and stable inflammatory biomarker and is calculated as the product of platelet and neutrophil counts divided by the lymphocyte count. PPN is used to assess local and systemic inflammation as well as the systemic immune response [10]. The PPN positively correlates with female bone mineral density [11] and infertility [12]. PLR is the ratio of platelets to lymphocytes and is related to the prognosis of breast cancer [13], hepatocellular carcinoma [14], and ampullary carcinoma [15]. SII, an emerging composite inflammatory index, is associated with poor outcomes in MM, suggesting its utility for monitoring inflammatory tumor microenvironments [16]. Emerging evidence indicates that SII plays a significant role in the onset, development, and progression of various cancers [17], including cervical cancer [18], gastric cancer [19], intrahepatic cholangiocarcinoma [20], and hepatocellular carcinoma [21]. However, their roles in the precursor state of MGUS remain unexplored. Furthermore, demographic and clinical heterogeneity in MGUS (e.g., a higher prevalence in older adults, males, and non-Hispanic Black individuals) raises questions regarding whether systemic inflammation interacts with these correlations to influence MGUS susceptibility.

Therefore, this study used extensive demographic, clinical, and laboratory data from the National Health and Nutrition Examination Survey (NHANES) to investigate the association between novel inflammatory indices, including PPN, PLR, SII, and CRP, and the prevalence of MGUS through a cross-sectional analysis, with the aim of providing new insights into disease management.

## Materials and methods

### Data source and population selection

**Fig. 1:**
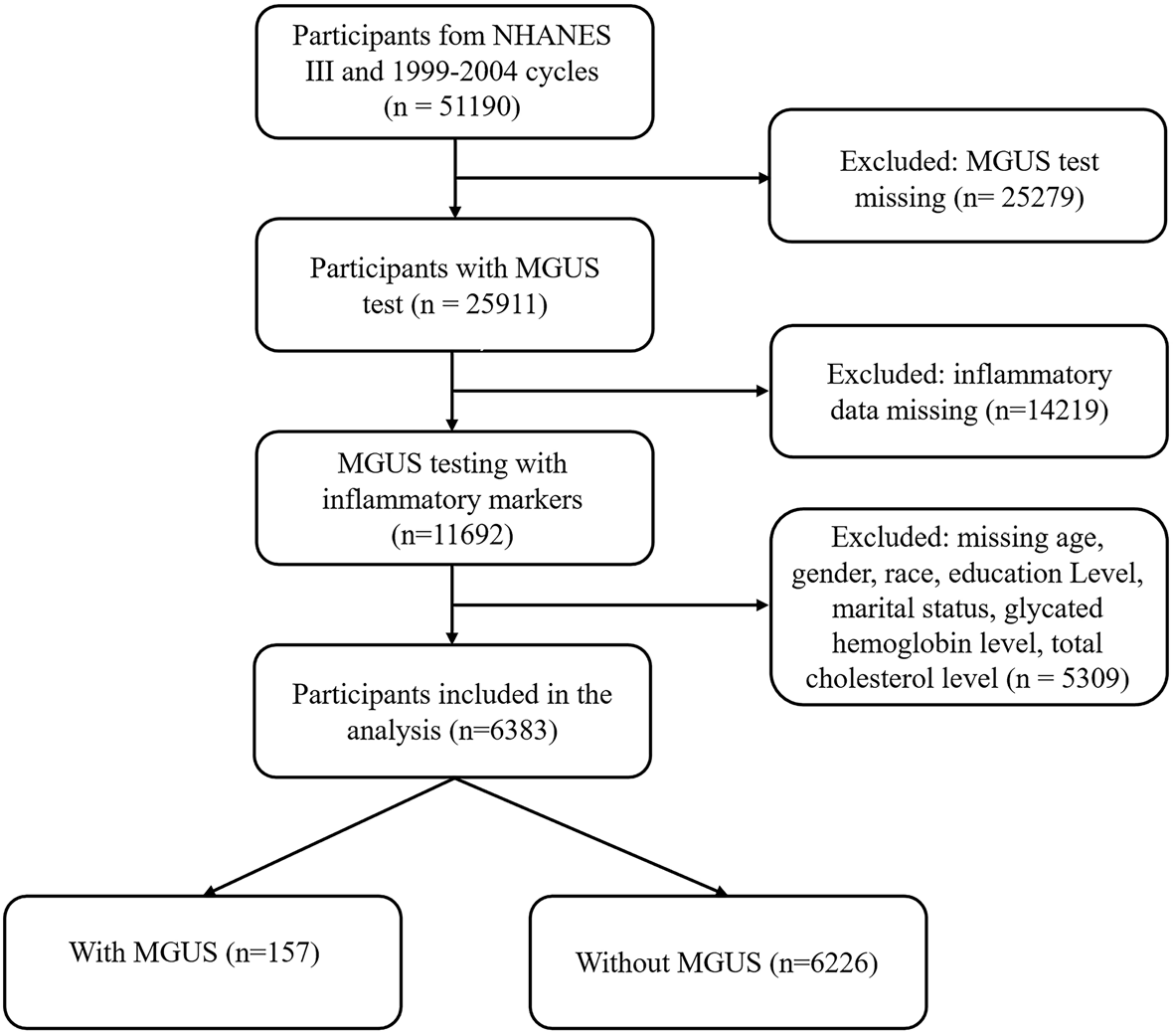
Participant selection flowchart for the NHANES analysis. The flowchart illustrates the inclusion and exclusion criteria used to select participants from the NHANES dataset. The study sample included 6,383 participants, of whom 157 were identified with MGUS. NHANES, National Health and Nutrition Examination Survey; MGUS, monoclonal gammopathy of undetermined significance

The National Health and Nutrition Examination Survey (NHANES) is a national cross-sector survey conducted annually by the National Center for Health Statistics (CDC/NCHS). The survey provides extensive data on demographics, socioeconomic status, diet, healthcare, laboratory test results, and overall health. Detailed information on the respondent recruitment, survey design, and data collection is available online. This nationally representative cross-sectional survey was conducted biennially using both questionnaires and physical examinations. For our analysis, we extracted data from 6,383 participants in NHANES III (1988–1994) and from the 1999 to 2004 cycle (Fig. 1). Participants in each NHANES cycle were selected using a stratified multistage probability sampling method. The survey employed a complex sampling plan to select a representative sample of the non-institutionalized U.S. civilian population, with oversampling of older adults, non-Hispanic Blacks, Mexican Americans, and other demographic groups. The NCHS Research Ethics Review Board approved the NHANES, and written informed consent was obtained from all participants.

### Assessment of MGUS

MGUS testing was conducted at the Protein Immunology Laboratory of the Mayo Clinic in Rochester, Minnesota, USA, using methods described in a previous study. Serum protein agarose gel electrophoresis was performed on all participants. Samples with uncertain or definitive M proteins were further analyzed by serum protein immunofixation electrophoresis and serum-free light chain detection. The MGUS subtype was determined based on immunofixation electrophoresis results. All tests and interpretations were conducted by personnel blinded to the demographics and other sample details.

### Analytic samples

Since immunofixation electrophoresis (IFE) and serum protein electrophoresis (SPE) were available for both the NHANES III and the 1999–2004 cycles, our study included 51,190 participants from these two datasets. We excluded participants who did not meet the following criteria: missing MGUS results (*n* = 25,279), missing inflammatory data (*n* = 14,219), or missing data on other covariates (*n* = 5,309). After applying these exclusions, 6,383 participants were included in the final analysis, of whom 157 participants had undergone MGUS. These 6,383 NHANES participants represent an estimated 49.87 million non-institutionalized older adults in the United States.

### Definition of inflammatory indices

Lymphocyte count (LC), platelet count (PC) and neutrophil count (NC), expressed as 1,000 cells/μL, were obtained from complete blood count (CBC) inflammatory indices analyses. These data were used to calculate various inflammatory indices as follows: PPN was calculated by multiplying the platelet count (× 10^9^/L) by the neutrophil count (× 10^9^/L); SII was calculated using the formula PC (× 10^9^/L) × NC (× 10^9^/L)/LC (× 10^9^/L); and PLR was defined as the ratio of platelet count (× 10^9^/L) to lymphocyte count (× 10^9^/L). Additionally, the CRP level (mg/L) was included as an inflammatory indicator.

### Covariates

Sociodemographic covariates included age, sex, race (Mexican American, non-Hispanic White, non-Hispanic Black, and others), marital status (married, widowed, divorced, separated, living with a partner, and never married), and educational level (categorized as high school, below, or above high school). Hypercholesterolemia was defined as total cholesterol level greater than 5.7 mmol/L, and diabetes was defined as glycated hemoglobin (HGB) level greater than 6.1%. Missing values for categorical covariates were excluded. Multiple interpolation (MI) was applied to continuous covariates to maintain the sample size and reduce selection bias. MI is an approach to compensate for missing data based on five replications and a chained equation method in the R-MI procedure to account for missing data on covariates.

### Statistical analysis

All statistical analyses were conducted using R software (version 4.1.3). We applied the Mobile Examination Center (MEC) exam weights provided by the NHANES to account for the complex survey sampling design, changes in design across survey cycles, and oversampling of specific subgroups. Continuous variables with a normal distribution were expressed as means ± standard deviation, while those without a normal distribution were summarized as means and interquartile ranges (IQR). Categorical variables were presented as percentages. For continuous variables, either the t-test or Mann–Whitney U test was used, and chi-square tests were applied to categorical variables. A *p*-value of 0.05 or less was considered statistically significant. We used both univariate and multivariate logistic regression models to examine the relationship between inflammatory indices and the likelihood of developing MGUS. Model 1 represents the crude association, which was adjusted for covariates; model 2 was adjusted for age, sex, race, and lymphocyte count; and model 3 was further adjusted for hypercholesterolemia and diabetes. Additionally, categorical analysis of PPN levels was conducted by categorizing participants into three groups based on the quintiles of PPN levels: a low group (1st to 2nd quintiles, < 177.00), a medium group (3rd to 4th quintile, 1057.40–11685.00), and a high group (5th quintile, ≥ 11,780.00). Multivariate weighted logistic regression models were used to calculate odds ratios (ORs) and confidence intervals (CIs) to assess the association between PPN levels and MGUS. Given the right-skewed distribution of PPN levels, they were naturally log-transformed (ln PPN) to evaluate the association between PPN levels (treated as a continuous variable) and MGUS. Similarly, the PLR was natural log-transformed (ln PLR) to normalize its right-skewed distribution before inclusion in the logistic regression models. Further stratified analysis was conducted to identify the variables that modified the association among the participants.

## Results

### Baseline characteristics of the study population

Among the 6,383 participants aged 20 years from the NHANES, 157 (2.46%) were identified as having MGUS. Baseline characteristics of the study participants stratified by MGUS status are summarized in Table 1. Significant differences were observed between participants with and without MGUS, particularly in terms of age distribution, sex, race/ethnicity, and prevalence of hypercholesterolemia. Participants with MGUS were more likely to be older (≥ 65 years, 66.88% vs. 48.43%, *p* < 0.001) and male (57.96% vs. 55.00%, *p* = 0.004). Differences were also observed in PPN levels (1,075 [94.4–29,602] vs. 1,258.55 [28.5–61,408], *p* = 0.002), PLR (115 [2.774–8 05] vs. 109.258 [0.79–732], *p* = 0.010), lymphocyte counts (2.1 [0.4–54] vs. 2.3 [0.4–89.7], *p* = 0.026), neutrophil counts (4.3 [0.9–76] vs. 4.7 [0.5–88], *p* = 0.004), and urine creatinine (10,431 [796–39600] vs. 9547 [442–60300], *p* = 0.013). However, no significant differences were noted in terms of educational level, marital status, or the prevalence of type 2 diabetes (T2D). Overall, the results highlight the key demographic and clinical differences between individuals with and without MGUS.

**Table 1:** Baseline characteristics of the study participants grouped by MGUS status in NHANES (*n* = 6383)

Characteristics	Participants			*P-*value
	Total	Without MGUS	With MGUS	
N	6383	6226	157	
Age				< 0.001
20–65	3263 (51.12)	3211 (51.57)	52 (33.12)	
> 65	3120 (48.88)	3015 (48.43)	105 (66.88)	
Sex				0.511
Male	3515 (55.07)	3424 (55.00)	91 (57.96)	
Female	2868 (44.93)	2802 (45.00)	66 (42.04)	
Race/ethnicity				0.004
Mexican American	1412 (22.12)	1387 (22.28)	25 (15.92)	
Non-Hispanic White	937 (14.68)	920 (14.78)	17 (10.83)	
Non-Hispanic Black	3188 (49.95)	3107 (49.90)	81 (51.59)	
Other	846 (13.25)	812 (13.04)	34 (21.66)	
Education Level				0.332
Below high school	1742 (27.29)	1705 (27.39)	37 (23.57)	
Above high school	4641 (72.71)	4521 (72.61)	120 (76.43)	
Marital Status				0.057
Married	3644 (57.09)	3556 (57.12)	88 (56.05)	
Widowed	1207 (18.91)	1172 (18.82)	35 (22.29)	
Divorced	636 (9.96)	613 (9.85)	23 (14.65)	
Separated	184 (2.88)	183 (2.94)	1 (0.64)	
Living with partner	528 (8.27)	521 (8.37)	7 (4.46)	
Never married	184 (2.88)	181 (2.91)	3 (1.91)	
Hypercholesterolemia				0.800
No	4188 (65.61)	4083 (65.58)	105 (66.88)	
Yes	2195 (34.39)	2143 (34.42)	52 (33.12)	
T2D				0.950
No	5212 (81.65)	5083 (81.64)	129 (82.17)	
Yes	1171 (18.35)	1143 (18.36)	26 (17.83)	
PPN	1254.4 (28.5–61,408)	1258.55 (28.5–61,408)	1075 (94.4–29,602)	0.002
PLR	109.429 (0.79–805)	109.258 (0.79–732)	115 (2.774–805)	0.010
SII	506.647 (11.875–11,700)	507.213 (11.875–11,700)	479.091 (29.5–3256)	0.277
CRP	0.26 (0.01–19.8)	0.26 (0.01–19.8)	0.28 (0.01–3.4)	0.389
White Blood Cell	6.8 (2.1–99.99)	6.8 (2.1–99.99)	6.7 (2.4–14.4)	0.263
Lymphocyte	2.3 (0.4–89.7)	2.3 (0.4–89.7)	2.1 (0.4–54)	0.026
Neutrophil	4.7 (0.5–88)	4.7 (0.5–88)	4.3 (0.9–76)	0.004
HGB	14.1 (4.95–19.5)	14.1 (4.95–19.5)	13.95 (9.5–18.4)	0.127
Platelet	254 (14.5–999.9)	254 (14.5–999.9)	245 (59–428)	0.081
Urine albumin	8.9 (0.2–16,920)	8.8 (0.2–16,920)	12.1 (0.4–4880)	0.893
Urine Creatinine	9547 (442–60,300)	9547 (442–60,300)	10,431 (796–39,600)	0.013
LDL	3.15 (0.52–16.266)	3.15 (0.52–16.266)	3.103 (1.24–5.77)	0.937

Mean ± SD for continuous variables: the *P* value was calculated by the weighted linear regression model; (%) for categorical variables: the *P* value was calculated by the weighted Chi-square test

*Abbreviations*: *CRP* C-reactive protein, *LDL* Low-Density Lipoprotein, *MGUS* Monoclonal gammopathy of undetermined significance, *PPN* product of platelet and neutrophil counts, *PLR* Platelet-to-Lymphocyte Ratio, *SII* Systemic immunity-inflammation, *T2D* type 2 diabetes, *HGB* Hemoglobin, *NHANES* National Health and Nutrition Examination Survey

Ln PPN levels were significantly associated with the PLR, SII, CRP level, white blood cell count, lymphocyte count, neutrophil count, HGB level, and peripheral platelet count (Table 2). Significant differences were observed among the three PPN subgroups (Low, Medium, and High). Participants in the low subgroup exhibited the highest PPN levels (17,043 [11,760–61,408]) and peripheral platelet counts (290 [148–999.9]), along with the lowest SII (590.391 [212.982–11,700]), CRP level (0.21 [0.1–19.8]), and HGB levels (13.65 [4.95–19.5]). Conversely, the high subgroup displayed the lowest PPN levels (744.6 [28.5–1,056.9]), peripheral platelet counts (220 [14.5–479]), and white blood cell counts (5.9 [2.4–31.5]). Trends were also observed for lymphocyte counts, which were highest in low (2.3 [0.4–89.7]) and lowest in high (1.8 [0.4–27.5]), and for neutrophil counts, which followed a similar pattern (Low: 5.4 [2.6–80.9]; High: 3.3 [0.5–72]). These results highlight the notable biological and hematological differences among PPN subgroups.

**Table 2:** Comparison of inflammation and hemogram in ln PPN subgroup of NHANES population

Characteristics	ln PPN level				*P*-value
	Total	Low (Q1-Q2, < 177.00)	Medium (Q3-Q4, 1057.40–11685.00)	High (Q5, ≥ 11,780.00)	
PPN	1254.4 (28.5–61,408)	17,043 (11,760–61408)	1531.2 (1057–11732.5)	744.6 (28.5–1056.9)	< 0.001
PLR	109.429 (0.79–805)	9.761 (5.17–650)	126.667 (0.79–732)	122.353 (0.853–805)	< 0.001
SII	506.647 (11.875–11,700)	590.391 (212.982–11,700)	645 (12.611–5264.4)	397.895 (11.875–1901.25)	< 0.001
CRP	0.26 (0.01–19.8)	0.21 (0.1–19.8)	0.33 (0.01–16)	0.23 (0.01–18.1)	< 0.001
White Blood Cell	6.8 (2.1–99.99)	6.9 (2.9–22.4)	7.9 (2.1–99.99)	5.9 (2.4–31.5)	< 0.001
Lymphocyte	2.3 (0.4–89.7)	30 (1.5–61)	2.3 (0.4–89.7)	1.8 (0.4–27.5)	< 0.001
Neutrophil	4.7 (0.5–88)	61 (17.5–88)	5.4 (2.6–80)	3.3 (0.5–72)	< 0.001
HGB	14.1 (4.95–19.5)	13.65 (4.95–19.5)	14.3 (6.4–19)	14.2 (8.9–19.2)	< 0.001
Peripheral platelet	254 (14.5–999.9)	290 (148–999.9)	278 (32–949)	220 (14.5–479)	< 0.001

Data are expressed as GM ± SE or frequency (percentage). Percentages, geometric means, SE, and cutoff points were weight-adjusted using the NHANES-specified sampling weights. For categorical variables, *p*-values were calculated using the Rao–Scott chi-square test, and for continuous variables, *p*-values were calculated using the Kruskal–Wallis H test (non-normal distribution)

*Abbreviations*: *CRP* C-reactive protein, *PPN* Platelet-to-lymphocyte Ratio, *PLR* Platelet-to-Lymphocyte Ratio, *SII* Systemic immune inflammation, *HGB* Hemoglobin, *SE* standard error, *NHANES* National Health and Nutrition Examination Survey

### The associations between inflammatory indices and MGUS

As shown in Table 3, ln PPN levels were significantly associated with MGUS. In Model 1, low ln PPN was significantly associated with MGUS (OR = 2.62; 95% CI: 1.54–4.75), while the medium PPN group had an OR of 2.20 (95% CI: 1.28–4.01). After adjusting for age, sex, race, and lymphocyte count (Model 2), the low and medium PPN groups had significantly higher odds of MGUS, with ORs of 2.27 (95% CI: 1.11–4.74) and 2.01 (95% CI: 1.04–3.97), respectively. These findings persisted in the fully adjusted model (model 3), which additionally accounted for hypercholesterolemia and diabetes (low OR = 2.26, medium OR = 2.01). Notably, the patients with MGUS have lower PPN values than that of those without MGUS (*p-trend* = 0.001). For ln PLR levels, no significant associations were observed across any category (all *p* > 0.05). In conclusion, ln PPN levels were independently associated with MGUS in this cross-sectional population, whereas ln PLR levels had no significant association.

**Table 3:** Odds ratios (95% CI) of MGUS and ln PPN levels in the NHANES follow-up study

Categories	MGUS			
	MGUS/observations (n/N)	Model 1	Model 2	Model 3
ln PPN level				
Low (Q1-Q2, < 177.00)	7 (305)	2.62 (1.54–4.75)^***^	2.27 (1.11–4.74)^*^	2.26 (1.11–4.72)^*^
Medium (Q3-Q4, 1057.40–11685.00)	11 (363)	2.20 (1.28–4.01)^**^	2.01 (1.04–3.97)^*^	2.01 (1.04–3.98)^*^
High (Q5, ≥ 11,780.00)	20 (332)	Ref	Ref	Ref
*P-*value for trend		0.001	0.08	0.08
ln PLR level				
Low (Q1-Q2, < 93.16)	15 (530)	Ref	Ref	Ref
Medium (Q2-Q3, 93.93–163.13)	16 (322)	1.60(1.11–2.31)^**^	1.30 (0.81–2.17)	1.31(0.81–2.16)
High (Q5, ≥ 163.50)	7 (148)	1.36(0.88–2.16)	1.07 (0.61–1.90)	1.07(0.61–1.90)
*P-*value for trend		0.07	0.97	0.97

ORs were estimated using multivariate logistic regression models and were weight-adjusted using NHANES-specified sampling weights. Model 1 was adjusted for no covariates. Model 2 was adjusted for age, sex, race, and lymphocyte count. Model 3 was further adjusted for hypercholesterolemia and diabetes

*MGUS* Monoclonal gammopathy of undetermined significance, *ln PPN* log-transformed product of platelet and neutrophil counts, *ln PLR* log-transformed platelet-to-lymphocyte ratio, *OR* odds ratio, *NHANES* National Health and Nutrition Examination Survey, *CI* confidence intervals

^*^p < 0.05, ^**^p < 0.01, ^***^p < 0.001

### Baseline characteristics of the ln PPN level subgroup

As shown in Table 4, the baseline characteristics of the participants based on the subgroups of PPN levels (Low, Medium, High) were significantly associated with age, race/ethnicity, education level, marital status, hypercholesterolemia, T2D, and incidence of MGUS. Additionally, significant differences in the distributions of age, race/ethnicity, educational level, marital status, hypercholesterolemia, T2D, and MGUS were observed among the three PPN subgroups.

**Table 4:** Baseline characteristics of participants based on subgroups of PPN levels in the NHANES follow-up study

Characteristics	PPN level				*P-*value
	Total	Low (Q1-Q2)	Medium (Q3-Q4)	High (Q5)	
N	6383	1277	2553	2553	
Age					< 0.001
20–65	3263 (51.12)	985 (77.13)	1229 (48.14)	1049 (41.09)	
> 65	3120 (48.88)	292 (22.87)	1324 (51.86)	1504 (58.91)	
Gender					0.511
Male		3515 (55.07)	3424 (55.00)	91 (57.96)	
Female		2868 (44.93)	2802 (45.00)	66 (42.04)	
Race/ethnicity					< 0.001
Mexican American	1412 (22.12)	307 (24.04)	559 (21.90)	546 (21.39)	
Non-Hispanic White	937 (14.68)	478 (37.43)	281 (11.01)	178 (6.97)	
Non-Hispanic Black	3188 (49.95)	438 (34.30)	1444 (56.56)	1306 (51.16)	
Other	846 (13.25)	54 (4.23)	269 (10.54)	523 (20.49)	
Education Level					0.020
Below grade 9	1742 (27.29)	379 (29.68)	653 (25.58)	710 (27.81)	
Above grade 9	4641 (72.71)	898 (70.32)	1900 (74.42)	1843 (72.19)	
Marital Status					< 0.001
Married	3644 (57.09)	686 (53.72)	1421 (55.66)	1537 (60.20)	
Widowed	1207 (18.91)	129 (10.10)	529 (20.72)	549 (21.50)	
Divorced	636 (9.96)	102 (7.99)	282 (11.05)	252 (9.87)	
Separated	184 (2.88)	55 (4.31)	80 (3.13)	49 (1.92)	
Living with partner	528 (8.27)	254 (19.89)	164 (6.42)	110 (4.31)	
Never married	184 (2.88)	51 (3.99)	77 (3.02)	56 (2.19)	
Hypercholesterolemia					0.011
No	4188 (65.61)	879 (68.83)	1633 (63.96)	1676 (65.65)	
Yes	2195 (34.39)	398 (31.17)	920 (36.04)	877 (34.35)	
T2D					< 0.001
No	5328 (83.47)	1140 (89.27)	2063 (80.81)	2125 (83.24)	
Yes	1055 (16.53)	137 (10.73)	490 (19.19)	428 (16.76)	
MGUS					0.002
No	6226 (97.54)	1262 (98.83)	2488 (97.45)	2476 (96.98)	
Yes	157 (2.46)	15 (1.17)	65 (2.55)	77 (3.02)	

Mean ± SD for continuous variables: the *P* value was calculated by the weighted linear regression model; (%) for categorical variables: the *P* value was calculated by the weighted Chi-square test

*Abbreviations*: *CRP* C-reactive protein, *LDL* Low-Density Lipoprotein, *MGUS* Monoclonal gammopathy of undetermined significance, *PPN* product of platelet and neutrophil counts, *PLR* Platelet-to-Lymphocyte Ratio, *SII* Systemic immune inflammation, *T2D* type 2 diabetes, *NHANES* National Health and Nutrition Examination Survey

Comparing the low and high level groups, the high level group exhibited the highest percentage of participants aged > 65 years, the highest prevalence of non-Hispanic Black participants, and a higher incidence of marital status. The high level group also showed a higher proportion of individuals with hypercholesterolemia and T2D, as well as a higher incidence of MGUS. Conversely, the low level group had a greater proportion of participants with education levels below grade 9.

These findings indicate that ln PPN levels are significantly associated with several baseline characteristics, highlighting the influence of age, race/ethnicity, and health conditions, such as hypercholesterolemia and T2D, on the distribution of PPN subgroups.

### Subgroup analysis

**Fig. 2:**
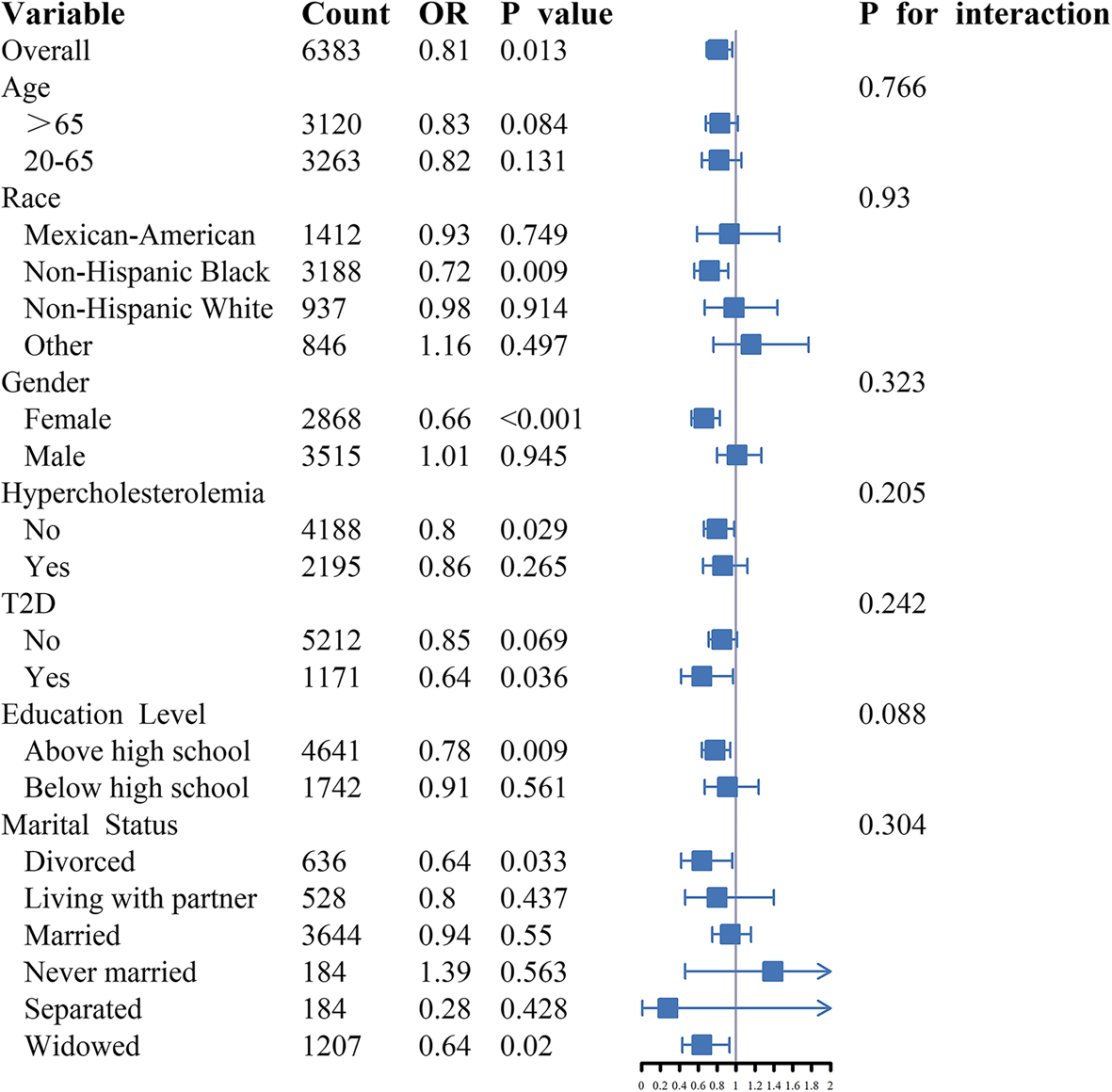
Subgroup analyses for the relationship between PPN and MGUS. The model was adjusted for age, race, sex, hypercholesterolemia, T2D, education level, marital status, and marital status. MGUS, monoclonal gammopathy of undetermined significance; PPN, platelet-neutrophil product; T2D, type 2 diabetes

To further investigate the potential association between lnPPN levels and MGUS, we stratified the participants into subgroups based on age, race, sex, hypercholesterolemia, T2D, education level, and marital status (Fig. 2). In the subgroup analysis, a statistically significant association was observed only in the overall population, females, Non-Hispanic Blacks, individuals without hypercholesterolemia, those without T2D, individuals with education levels above high school, and divorced or widowed participants (all *p* < 0.05). However, no significant interactions were found between lnPPN levels and MGUS across the subgroups (all *p values* for interaction > 0.05).

## Discussion

MGUS is a prevalent and typically asymptomatic immunoglobulin disorder, characterized by the presence of monoclonal immunoglobulins in the serum. Although generally considered benign, MGUS may serve as a precursor to MM and other plasma cell-related disorders [22]. The transformation of MGUS into multiple myeloma or other malignant plasma cell disorders, such as light-chain amyloidosis and monoclonal immunoglobulin deposition, is a key clinical concern [23]. Transformation in patients with MGUS is associated with factors such as the level of M-protein, proportion of plasma cells in the bone marrow, and other clinical factors, including family history, age, and sex [24]. The prevalence of MGUS increases with age, particularly in individuals over 60 years old. Studies have reported an overall prevalence of approximately 3–4%, with a significantly higher prevalence in individuals aged 50 years and older [3]. Research conducted in the United States indicate that the prevalence of MGUS in individuals over 60 years of age may be as high as 5–7%. Furthermore, the prevalence of MGUS varies across racial and geographic populations [25].

Inflammation plays a pivotal role in tumorigenesis and tumor progression. Cancer, stromal, and immune cells form a complex interactive network by establishing a dynamic inflammatory tumor microenvironment. These cells exhibit significant plasticity and work synergistically to promote the entire tumorigenic process from initiation and growth to metastasis [26]. The relationship between inflammation and MGUS has been studied extensively. Although it is generally regarded as a benign immune disorder, accumulating evidence indicates that chronic inflammation may play a critical role in the initiation, progression, and transformation of MGUS into malignant plasma cell disorders, including multiple myeloma. Inflammatory cytokines, including TNF-α, IL-6, and IL-1β, are closely associated with plasma cell growth and differentiation. Immune regulation may be disrupted in inflammatory environments, leading to abnormal plasma cell proliferation and excessive immunoglobulin production [7]. Reportedly, chronic inflammation can accelerate disease progression by promoting immune suppression, inhibiting the function of tumor suppressors, and activating tumor-associated signaling pathways such as the NF-κB pathway, particularly within the BMME [8, 9]. Additionally, inflammation may provide a favorable microenvironment for the growth of malignant plasma cells by modulating the function of immunosuppressive cells (such as regulatory T cells and myeloid-derived suppressor cells), influencing immune evasion mechanisms, and enhancing the supportive role of BMME [27].

Inflammatory markers have consistently been shown to be strongly associated with a poor prognosis in conditions such as hypertension, acute myocardial infarction, ovarian cancer, diabetic nephropathy, and Parkinson’s disease [28]. Recent studies have confirmed that the inflammatory index positively correlates with disease [29]. This study provides novel insights into the association between PPN and the prevalence of MGUS in a large cohort using the NHANES dataset. Our findings revealed a significant correlation between low PPN levels and the development of MGUS (OR = 2.26 for the low PPN group), whereas no significant association was observed for PLR. Additionally, subgroup analysis demonstrated that this association was particularly pronounced in females, non-Hispanic Black individuals, and those without metabolic diseases. Analysis of baseline characteristics further indicated that PPN levels were significantly associated with age, race, and educational background, suggesting that chronic inflammation may be modulated by various sociobiological factors. Interestingly, in contrast to the traditionally high level of inflammation associated with disease progression and poor prognosis, the findings of this study on MGUS showed significant differences. We found that patients with MGUS have lower PPN values. This difference may reflect the fact that the pathophysiology of MGUS does not involve accumulation of simple proinflammatory signals. We look forward to future studies to address this question. Moreover, to the best of our knowledge, this study is the first to validate the association between the PPN and MGUS in a large population cohort, providing new evidence for the application of composite inflammation indices in precursor diseases. Although similar studies have been conducted on MM, the application of the PPN in MGUS remains unprecedented, offering fresh perspectives on the pathophysiology of the disease.

Our study has several advantages. This is the first study to explore the relationship between the PPN index and MGUS based on a large sample size of the NHANES database. Furthermore, we used a weighted logistic regression model for analysis because the NHANES database is composed of multi-stage complex sampling data and we adjusted for other covariates, which makes the conclusions drawn in this study more accurate and reliable. Although the trend test results were not statistically significant after adjusting for the covariates, the correlation between the two remained significant. Additionally, we transformed the PPN before the analysis to ensure that it had a normal distribution. Finally, subgroup analysis was used to further stratify the data.

However, this study also had some limitations. Fundamental to our study design, the cross-sectional nature of the NHANES data precluded any causal inference between PPN levels and MGUS. While our analyses were adjusted for key confounders, the temporal sequence of PPN changes relative to MGUS onset remains indeterminate; PPN could theoretically be either a driver or consequence of early clonal expansion. This ambiguity is an inherent constraint of population-based surveys that lack a longitudinal follow-up. Future longitudinal studies must determine whether PPN is a cause of MGUS or both, or whether it is a large-scale outcome. Additionally, the relatively low prevalence of MGUS (2.46%) may limit the power of the subgroup analyses, particularly in certain racial groups with insufficient sample sizes. Unmeasured confounding variables (e.g., genetic variations and environmental exposure) could also have affected the results. Finally, since NHANES did not record some classic inflammatory factors (such as TNF-α, IL-6, IL-10, etc.), relevant indicators cannot be included to obtain more comprehensive results. More researchers should continue to explore the inflammatory markers in MGUS. We hope that this study will provide a scientific reference for future research. We will also include more classical inflammatory indices in future studies to explore the relationship between MGUS and PPN indices.

## Conclusion

To the best of our knowledge, this is the first study to reveal a significant correlation between PPN levels and MGUS prevalence, suggesting that chronic inflammation plays a crucial role in the development of precursor diseases. This outcome compensated for previous studies and offered new directions for the stratification and mechanistic studies of MGUS. Future longitudinal studies and experimental models are needed to explore the role of inflammatory factors in plasma cell colony regulation, ultimately paving the way for the development of precise preventive strategies.

## Data Availability

The datasets generated during and/or analysed during the current study are available in the NHANES repository, (https://www.cdc.gov/nchs/nhanes/).

## Abbreviations

CRP: C-reactive protein
LC: Lymphocyte count
MGUS: Monoclonal gammopathy of undetermined significance
MM: Multiple myeloma
NC: Neutrophil count
NHANES: National Health and Nutrition Examination Survey
PC: Platelet count
PLR: Platelet-to-lymphocyte ratio
PPN: Product of platelet and neutrophil count
SII: Systemic immune inflammation index
T2D: Type 2 Diabetes
